# An active new formulation of iron carried by aspartyl casein for iron-deficiency anemia: results of the ACCESS trial

**DOI:** 10.1007/s00277-023-05197-3

**Published:** 2023-04-06

**Authors:** Maria Tsilika, John Mitrou, Nikolaos Antonakos, Ioulia K. Tseti, Georgia Damoraki, Konstantinos Leventogiannis, Evangelos J. Giamarellos-Bourboulis

**Affiliations:** 1 4th Department of Internal Medicine, Medical School, National and Kapodistrian University of Athens, Athens, Greece; 2 UNI-PHARMA SA, Kifissia, Greece; 3 4th Department of Internal Medicine, ATTIKON University General Hospital, 1 Rimini Street, 124 62 Athens, Greece

**Keywords:** Iron deficiency anemia, Iron sulfate, Casein, Hemoglobin, Physical signs

## Abstract

Oral iron supplementation is the cornerstone for the management of iron-deficiency anemia. A new oral formulation of iron conjugated with N-aspartyl-casein (Fe-ASP) (Omalin®, Uni-Pharma) is studied in the ACCESS double-blind, double-dummy randomized clinical trial; 60 patients were randomized to 12-week oral treatment twice every day either with oral ferrous sulfate (FeSO_4_) delivering 47 mg elementary iron or oral Fe-ASP delivering 40 mg elementary iron. Participants had hemoglobin less than 10 g/dl, decreased red blood cell (RBC) count, and ferritin lower than 30 ng/ml; patients with a medical history of malignancy were excluded. The primary endpoint was the increase of Hb in the first 4 weeks of treatment, and the study was powered for non-inferiority. A new score of global improvement was introduced where all participants were given one point for any at least 10% increase of Hb, RBC, and reticulocytes. At week 4, the mean (SE) change of Hb was 0.76 g/dl in the FeSO_4_ group and 0.83 g/dl in the Fe-ASP group (*p*: 0.876). The odds for worse allocation of the global score were 0.35 in the Fe-ASP group compared to the FeSO_4_ group. Patients in the Fe-ASP group experienced a significant decrease in the number of IDA-related physical signs by week 4. No differences were found between the two groups in any of the patient-reported outcomes of fatigue and of gastrointestinal adverse events either at week 4 or at week 12. ACCESS is the most recent clinical trial showing the non-inferiority of Fe-ASP to FeSO_4_ for the primary endpoint of the Hb change.

**Supplementary Information:**

The online version contains supplementary material available at 10.1007/s00277-023-05197-3.

## Introduction

Despite the undeniable progress in Western societies, iron deficiency anemia (IDA) remains the most common cause of anemia with 8.18, 8.93, 12.42, and 14.14 incident cases per 1000 person-years in Belgium, Italy, Germany, and Spain repectively [1]. IDA develops as a result of increased iron requirements (usually happening during infancy, childhood, adolescence, and pregnancy), low iron intake (such as malnutrition or a vegan diet), decreased intestinal absorption of iron (usually after gastrectomy, duodenal bypass and bariatric surgery, infection by *Helicobacter pylori*, and intake of proton pump inhibitors and H_2_ blockers), chronic blood loss (such as gastrointestinal and uterine neoplasms and intake of anticoagulants), chronic kidney disease, chronic systolic heart failure, and inflammatory bowel disease (IBD), postoperatively [2]. Iron deficiency and IDA have deleterious effects. IDA is an independent comorbidity associated with a 1.88 hazard ratio for death in patients with chronic heart failure [3]. Among elderly patients, those with hemoglobin less than 12 g/dl have a 3-fold greater risk for death [4]. Anemia is also increasing by an average of 65% the risk of death among cancer patients [5]. IDA in pregnancy increases the odds of premature birth [6]; it is associated with prolongation of the QRS and the QTc interval in the electrocardiogram [7]; and it affects children’s immune function of the neutrophils [8].

The deleterious effect of IDA makes early recognition and management mandatory. The cornerstones of management are recognition of the cause of iron deficiency or malabsorption, management of the cause of iron loss and efficient iron supplementation. Indeed, appropriate replacement of iron storage through parenteral or enteral iron supplementation improves the quality of life in cancer patients [5] and decreases the odds of death in patients with chronic heart failure [9]. In patients with cancer and chronic heart failure, most of the clinical benefit is coming after the administration of intravenous iron formulations [5, 9] probably because oral formulations are hampered by limited intestinal absorption [10]. The main available oral iron preparations for iron supplementation are ferrous sulfate (FeSO_4_), ferrous gluconate, and ferrous fumarate. The main limitations of treatment are gastrointestinal (GI) side effects observed in almost 40% of cases. These are gastric discomfort, nausea, vomiting, and constipation, and they are caused due to the oxidation of ferrous irons in the stomach by acidic gastric fluid into insoluble salts. A recent meta-analysis of 43 trials showed that the cumulative odds ratio (OR) for the GI side effects with the use of preparations of FeSO_4_ ranged between 2.43 and 3.05 [11]. The limited intestinal absorption of oral iron preparations leads to modulation of the composition of the gut microbiome. Two randomized controlled trials (RCT) in infants from Africa showed that the consumption of iron-enriched micronutrient powders was associated with the acquisition of pathogenic enterobacteria in the gut flora [12].

A formulation of iron conjugated to one *N*-acetyl-aspartylated derivative of casein (Fe-ASP) has recently been developed. Due to the casein coating, it is anticipated that iron is converted to a smaller extent in the stomach into insoluble salts. In this way, more iron reaches the duodenum to become absorbed whereas GI side effects are less often [13]. ACCESS (ferrous ACetyl-aspartylated Casein formulation Evaluation over ferrouS Sulfate in iron deficiency anemia) is a randomized clinical trial aiming to show that the new oral formulation of Fe-ASP is non-inferior to oral FeSO_4_ for the restoration of hemoglobin (Hb) in IDA. The effect on the symptoms of anemia and on biomarkers of iron deficiency and the incidence of GI tract side effects are also investigated.

## Patients and methods

ACCESS is a double-dummy, double-blind, randomized, and phase IV clinical trial which took place in the 4th Department of Internal Medicine of the ATTIKON University General Hospital. The study was approved by the Ethics Committees of ATTIKON hospital (approval 2596/31-10-2017), by the National Ethics Committee of Greece (approval 93/17) and by the National Organization for Medicines of Greece (approval IS091/17). Written informed consent was obtained from all study participants before screening. The study was registered (EudraCT number: 2017-002972-5; ClinicalTrials.gov NCT03524651)

Enrolled patients were adults of either gender who provided written informed consent and who were meeting all following criteria, also considered by others to frame the characteristics of IDA [14-16]: (i) Hb below 10g/dl, (ii) absolute red blood cell (RBC) count below 4.7 × 10 [6]/mm^3^ for men and below 4.2 × 10 [6]/mm^3^ for women, (iii) mean corpuscular volume (MCV) of RBCs below 80 fl, (iv) mean corpuscular Hb (MCH) of RBCs below 27 pg, and (v) total ferritin below 30 ng/ml. Exclusion criteria were as follows: history of acute myelogenous or lymphoblastic leukemia, history of multiple myeloma, history of primary or secondary myelodysplastic syndrome, planning for start of chemotherapy or radiotherapy within the next 30 days, active or planned treatment with recombinant human erythropoietin, intake of chemotherapy or radiotherapy the last six months, history of hemochromatosis, history of celiac disease, liver cirrhosis of Child-Pugh stage II or III, any active overt bleeding, and pregnancy or lactation.

Screening was done against all exclusion criteria. If none was met, then the absolute RBC count, Hb, MCV, MCH, and ferritin were analyzed. If the obtained values for all these parameters were within the inclusion criteria, then the patient could be enrolled in the study. The total screening period could not last for more than 1 week.

Patients were randomly 1:1 assigned to double-blind and double-dummy treatment with FeSO_4_ or Fe-ASP every 12 h for 12 weeks. Randomization was provided by a sealed envelope to the investigators; the randomization sheet was generated by the study Sponsor. The investigator and patient were blind to the allocated intervention. Following randomization, every patient was delivered two different boxes, one for each month of treatment. The first box contained capsules with a three-digit number on the outside. The second box contained vials with a four-digit number on the outside. The allocated numbers were provided inside the sealed envelopes. Every patient was randomized into one of the following groups as follows:

- FeSO_4_: Patients were taking every day for 12 weeks two oral capsules of 150 mg ferrous sulfate delivering 47 mg of active elementary iron. The capsules had the following excipients: polyvidone K30, titanium dioxide CI 77891 E171, glyceryl monostearate, beeswax white, sucrose, iron oxide red CI 77491 E172, green lake 180790, starch maize, talc purified, kaolin heavy, erythrosine CI 45430 E127, patent blue V CI 42051 E131, gelatin. Patients were instructed to receive the capsules either 2 h before meal or 2 h after meal. The same patients were also taking every day on exactly the same time for 12 weeks two placebo vials of 15 ml volume with excipients contained in the commercially available formulation Fe-Asp Omalin® (Uni-Pharma SA): sorbitol, propylene glycol, methyl parahydroxybenzoate, propylparaben sodium, water purified, flavor toffee.
- Fe-ASP: Patients were taking every day for 12 weeks two oral placebo capsules. The capsules were containing the following inactive excipients: polyvidone K30, titanium dioxide CI 77891 E171, glyceryl monostearate, beeswax white, sucrose, iron oxide red CI 77491 E172, green lake 180790, starch maize, talc purified, kaolin heavy, erythrosine CI 45430 E127, patent blue V CI 42051 E131, gelatin. Patients were instructed to receive the capsules either 2 h before meal or 2 h after meal. The same patients were taking every day at the same time for 12 weeks two vials of 15 ml volume of the Fe-Asp preparation Omalin® (Uni-Pharma SA) delivering 40 mg of elementary iron.

Every participant was subject to four study visits: at baseline (time 0), at 7 ± 1 days from the start of the study drug, at 4 weeks from the start of the study drug, and at 12 weeks from the start of the study drug. On visit days of baseline, 4 weeks, and 12 weeks, patients were (i) subject to complete physical examination with emphasis on the number of IDA-related findings, namely skin pallor, pallor at the conjunctiva, glossitis, cheilitis, koilonychia, splenomegaly, hepatomegaly, and enlarged lymph nodes. These eight signs were always evaluated by the same two evaluators: (ii) asked to grade the intensity of fatigue at a scale of 0 to 100mm. Patients were informed that 0 refers to the absence of fatigue, 1 to 30 to mild fatigue, 30 to 60 to moderate fatigue, and more than 60 to severe fatigue and that at each range of fatigue, he/she had to remember that the upper limit was the worst of the precise fatigue subcategory he/she had ever experienced; (iii) recording of any treatment-emergent adverse event (TEAE) with special emphasis given on the intensity of gastric discomfort, nausea, vomiting, and constipation. Patients were asked to grade each of these symptoms on a scale of 0 to 100mm. Patients were informed that 0 refers to the absence of the symptom and 100 to the worst intensity of this symptom they ever felt. On study visits of 7 days, 3ml of blood was collected after venipuncture of one forearm vein under aseptic conditions and collected into one EDTA-coated tube for absolute RCB counting, determination of the absolute reticulocyte count, and determination of MCV and MCH. On study visits of week 4 and week 12, 6 ml of blood was collected after venipuncture of one forearm vein under aseptic conditions; three milliliters is collected into one EDTA-coated tube for absolute blood cell counting, determination of the absolute reticulocyte count, and determination of MCV and MCH. Another 3 ml was collected into one pyrogen-free tube for measurements of ferritin and hepcidin. These two tubes are transported into the central lab. In blood collected at weeks 4 and 12, measurements of ferritin and hepcidin were done by enzyme immunosorbent assays (ferritin: ORGENTEC Diagnostika GmbH, Mainz, Germany; lower detection limit 75ng/ml; hepcidin Cloud-Clone Corp, Katy, TX, USA; lower detection limit 63 pg/ml).

The primary study endpoint was the non-inferiority of the increase of baseline Hb in the FeSO_4_ and in the Fe-ASP groups after the first 4 weeks of treatment.

The secondary study endpoints were the differences between the two groups of treatment in absolute reticulocyte count, absolute RBC count, Hb, MCV, and MCH; change of the fatigue symptoms; change of physical findings of IDA; circulating hepcidin and ferritin; and in the incidence of GI side effects. Since the daily amount of elementary iron delivered with the ferrous sulfate regimen was 94 mg and with the Fe-ASP regimen 80 mg, the increase of baseline Hb was adjusted per mg of delivered elementary iron.

The study was powered for the primary endpoint considering that the mean baseline increase of Hb after 4 weeks of treatment with the formulation of FeSO_4_ would be 0.7 g/dl^17^. With the assumption that there will be no difference between the two groups in the primary study endpoint and that the mean difference between the two groups will not be larger than 0.5 g/dl with a standard deviation of 0.6 g/dl, to demonstrate non-inferiority between the two groups of treatment with 80% power at the 5% level of significance, 25 patients should be enrolled in each arm. After adjustment for missing values, it was decided to enroll 30 patients in each arm.

Comparisons of quantitative characteristics between groups were done by the Student’s “t-test” and of qualitative characteristics by the Fisher’s exact test. The analysis was done for the intent-to-treat (ITT) population and for the per-protocol (PP) population for week 4 and for actual patients for week 12. The ITT population comprised all randomized patients. The PP population consisted of patients who did not prematurely stop the study drug and who were not in need of intravenous iron supplementation before the completion of week 4 of follow-up. The absolute and relative changes of each hematology index from baseline were calculated and expressed as means ±SE; comparisons were done by the Student’s *t*-test. The absolute and relative changes in the number of IDA-related findings from the baseline were also calculated and compared. In week 4, a global improvement score was introduced for each participant where one point was given for each 10% increase of Hb, of the absolute RBC count, and of the reticulocyte count from baseline. The score could get from 0 to 3 points, and comparisons between groups were done by ordinal regression analysis. Any *p*-value below 0.05 was considered significant.

## Results

**Fig. 1:**
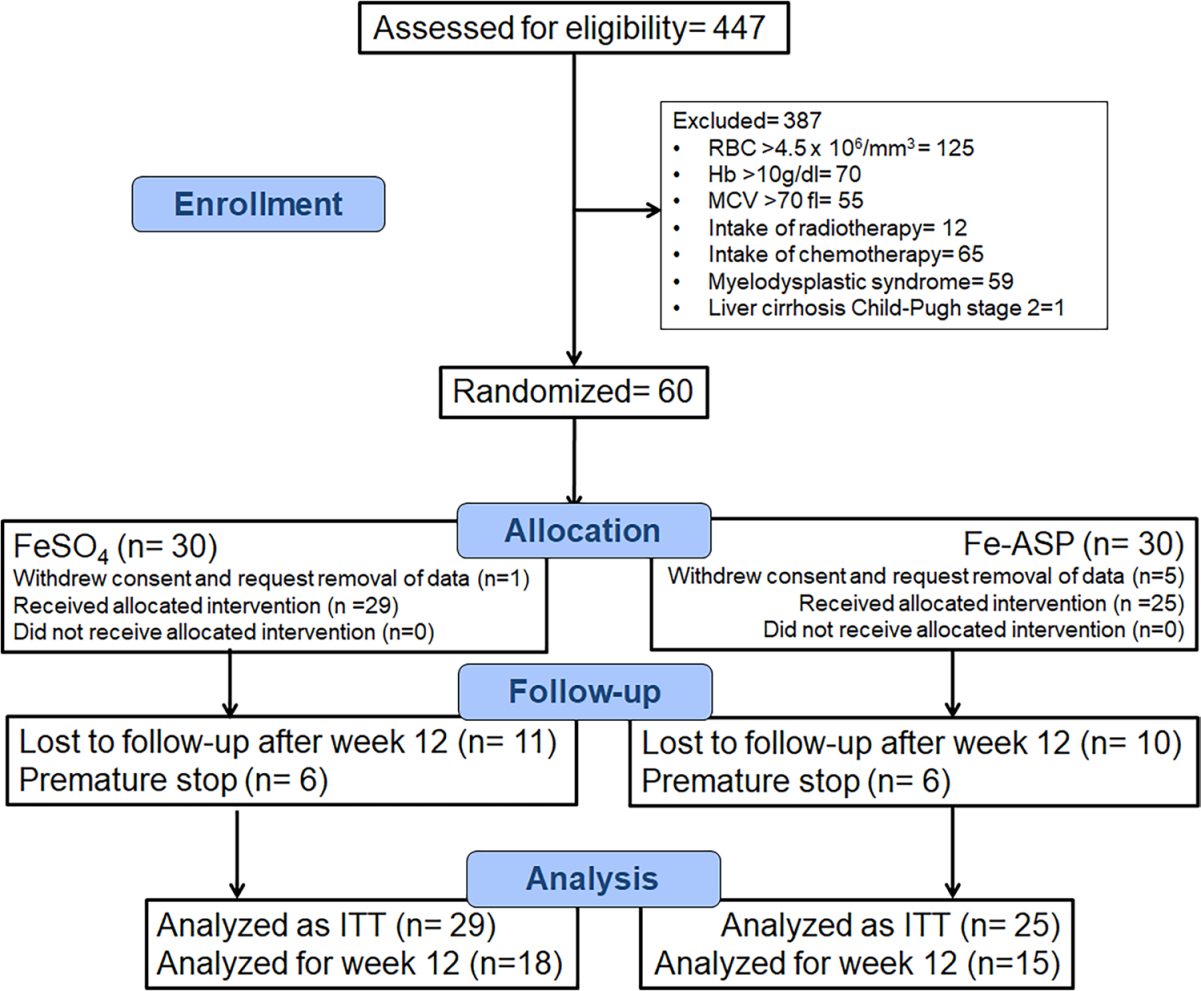
Study flow chart. Abbreviations: Fe-ASP, iron conjugated to *N*-acetyl-aspartylated derivative of Casein; FeSO_4_, ferrous sulfate; ITT, intent-to-treat

The first patient was enrolled on 21 May 2018, and the last visit of the last patient was on 09 October 2020. From 447 patients screened for eligibility, 60 patients were randomized; 30 in the FeSO_4_ group and 30 in the Fe-ASP group; one and five patients respectively withdrew consent and request removal of data leaving 29 and 25 patients respectively in the ITT population for the analysis of the primary endpoint (Table 1). Six patients in the FeSO_4_ group and six patients in the Fe-ASP group stopped the study drug prematurely due to lack of efficacy and were subject to intravenous iron administration. In total, 11 patients and 10 patients were lost at follow-up between weeks 4 and 12 leaving 18 patients and 15 patients respectively to be analyzed for efficacy by week 12 (Fig. 1).

**Table 1:** Baseline characteristics of enrolled patients

	FeSO_4_ (*n*= 29)	Fe-ASP (*n*= 25)	*p*-value
Female gender, *n* (%)	19 (65.5)	14 (56.0)	0.579
Age (years), mean (SD)	62.5 (16.1)	62.7 (21.2)	0.969
Charlson’s comorbidity index, mean (SD)	3.25 (2.66)	3.93 (3.53)	0.436
Red blood cell count (×10^6^/mm^3^), mean (SD)	3.94 (0.46)	3.83 (0.38)	0.472
Hematocrtit (%), mean (SD)	27.8 (2.9)	27.7 (3.1)	0.873
Hemoglobin (g/dl), mean (SD)	8.4 (1.08)	8.4 (0.9)	0.851
MCV (fl), mean (SD)	71.0 (7.6)	70.9 (6.6)	0.976
MCH (pg), mean (SD)	21.7 (3.3)	21.8 (2.4)	0.951
% reticulocytes	0.04 (0.09)	0.03 (0.02)	0.208
Absolute reticulocyte count (/mm^3^), mean (SD)	1451.8 (590.1)	1352.9 (644.8)	0.408
White blood cell count (/mm^3^), mean (SD)	7877.9 (2019.3)	7379.6 (3081.9)	0.480
Neutrophils (/mm^3^), mean (SD)	5209.1 (1479.5)	4465.0 (1602.2)	0.082
Lymphocytes (/mm^3^), mean (SD)	1856.0 (761.4)	2114.5 (1693.4)	0.462
Monocytes (/mm^3^), mean (SD)	602.9 (221.9)	586.9 (227.8)	0.794
Platelet count (×10^3^/mm^3^), mean (SD)	355.2 (118.4)	301.3 (124.3)	0.109
Ferritin (ng/ml), mean (SD)	11.9 (5.7)	12.4 (5.6)	0.436
Hepcidin (pg/ml), median (IQR)	<63.0 (9.0)	< 63 (0)	1.00
Intensity of fatigue (mm), mean (SD)	52.4 (29.4)	68.4 (21.9)	0.030
Number of physical signs of iron-deficiency, *n* (%)	1.37 (1.26)	2.08 (1.18)	0.041
Physical signs of iron-deficiency, *n* (%)			
Skin pallor	16 (55.2)	20 (80.0)	0.082
Pallor on conjunctiva	15 (51.7)	18 (72.0)	0.166
Glossitis	4 (13.8)	3 (12.0)	1.00
Chelitis	3 (10.3)	4 (16.0)	0.692
Koilonychia	1 (3.4)	1 (4.0)	1.00
Hepatomegaly	1 (3.4)	3 (12.0)	0.326
Enlarged cervical lymph nodes	9 (0)	3 (12.0)	0.093
Co-morbidities, *n* (%)			
Type 2 diabetes mellitus	11 (37.9)	7 (28.0)	0.565
Chronic heart failure	4 (13.8)	6 (24.0)	0.485
Chronic obstructive pulmonary disease	1 (3.4)	6 (24.0)	0.041
Chronic renal disease	3 (10.3)	5 (20.0)	0.449
Stroke	4 (13.8)	2 (8.0)	0.675
Coronary heart disease	3 (10.3)	5 (20.0)	0.449
Atrial fibrillation	1 (3.4)	3 (12.0)	0.326
Hip surgery	2 (6.9)	2 (8.0)	1.00
Predisposing factors, *n* (%)			
Peptic ulcer	4 (13.8)	1 (4.0)	0.358
*Helicobacter pylori* gastritis	2 (6.9)	1 (4.0)	1.00
Uterine myomas	1 (3.4)	1 (4.0)	1.00
Colon polyps	1 (3.4)	1 (4.0)	1.00

Abbreviations: *IQR*, interquartile range; *MCH*, mean corpuscular hemoglobin; *MCV*, mean corpuscular volume; *SD*, standard deviation

**Fig. 2:**
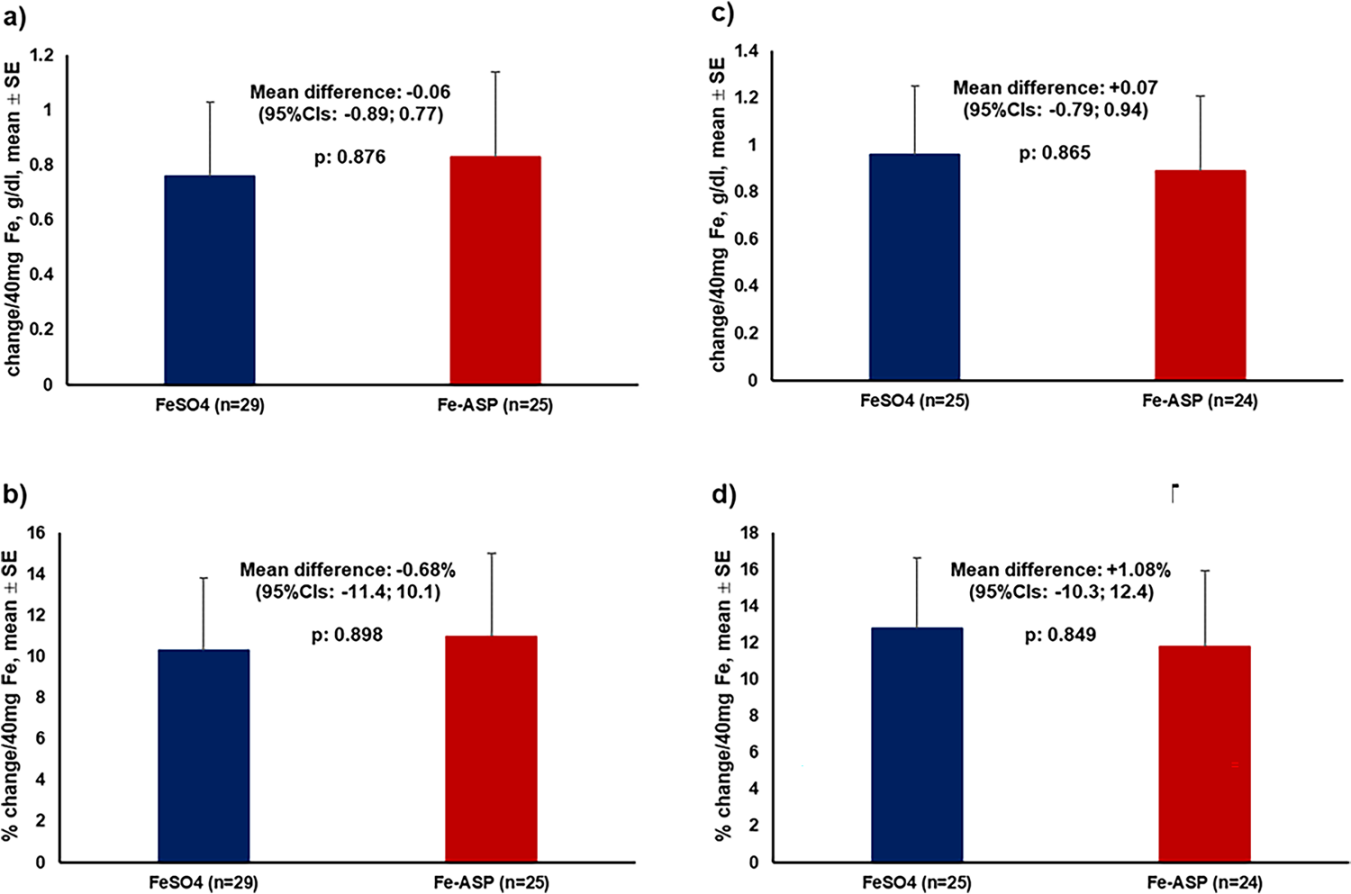
Primary study endpoint by week 4. The analysis is presented separately for the intent-to-treat (ITT) population and for the per-protocol population (PP). **a** and **b** Represent the absolute and relative changes of baseline hemoglobin (Hb) under treatment with capsules of ferrous sulfate (FeSO_4_) and iron aspartyl-casein (Fe-ASP) for the ITT population. **c** and **d** Represent the absolute and relative changes of baseline Hb under treatment with capsules of FeSO_4_ and Fe-ASP for the PP population. The confidence intervals (CI) of non-inferiority are provided. The PP population consisted of patients who did not prematurely stop the study drug and who were not in need of intravenous iron supplementation before the completion of week 4 of follow-up

The primary study endpoint was successful for non-inferiority between the two groups of treatment in both the absolute and relative change of baseline Hb by week 4 (Fig. 2a and 2b). As mentioned above, six patients of each group stopped the study drug prematurely due to lack of efficacy and were subject to intravenous iron administration. The median time to this event was 15 days in the FeSO_4_ group and 61.5 days in the Fe-ASP group (*p*: 0.045). Analysis in the PP population confirmed the non-inferior result of the primary endpoint in the ITT population (Fig. 2c and 2d). Based on the achieved results, one *post hoc* power calculation was run. This was based on the standard deviation of the primary endpoint for the absolute change of Hb from baseline at week 4 which was 1.49 g/dl and on the −0.06 g/dl observed difference in the means between the two treatment groups. The number of required subjects per arm to prove non-inferiority with 80% power at the 5% level of significance was 34 per arm, i.e., close to the enrolled subjects in the trial.

**Fig. 3:**
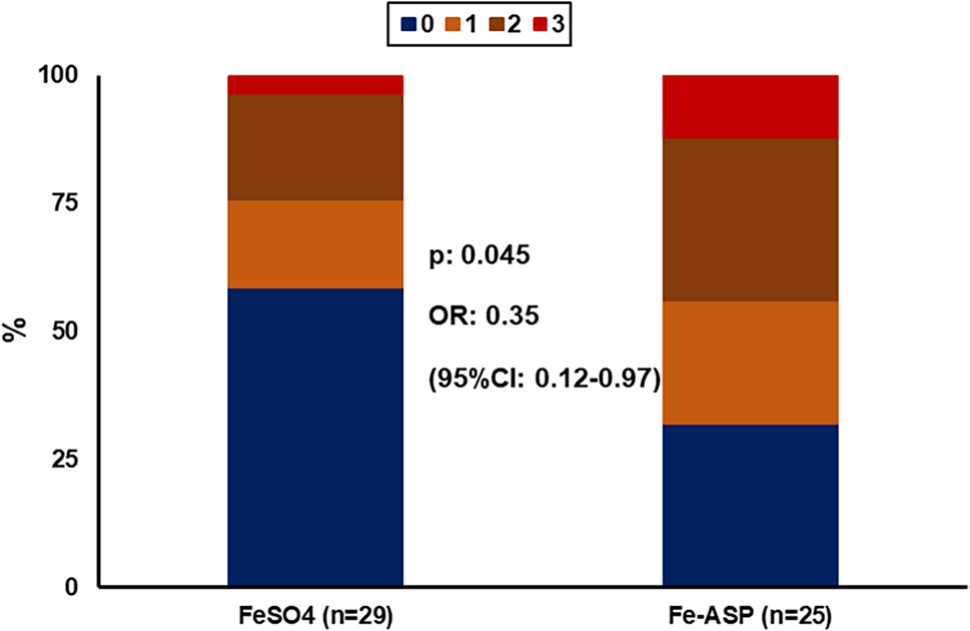
Ordinal regression analysis of the global evaluation score of patients. In this score, every participant scores one point for each 10% increase from the baseline of total red blood cell count, hemoglobin, and total reticulocyte count. The assumptions of the ordinal regression were the Goodness-of-fit test with a *p*-value of 0.861, and the test of parallel lines with a *p*-value of 0.861. Abbreviations: CI, confidence intervals; Fe-ASP, iron conjugated to *N*-acetyl-aspartylated derivative of Casein; FeSO_4_, ferrous sulfate; OR, odds ratio

**Fig. 4:**
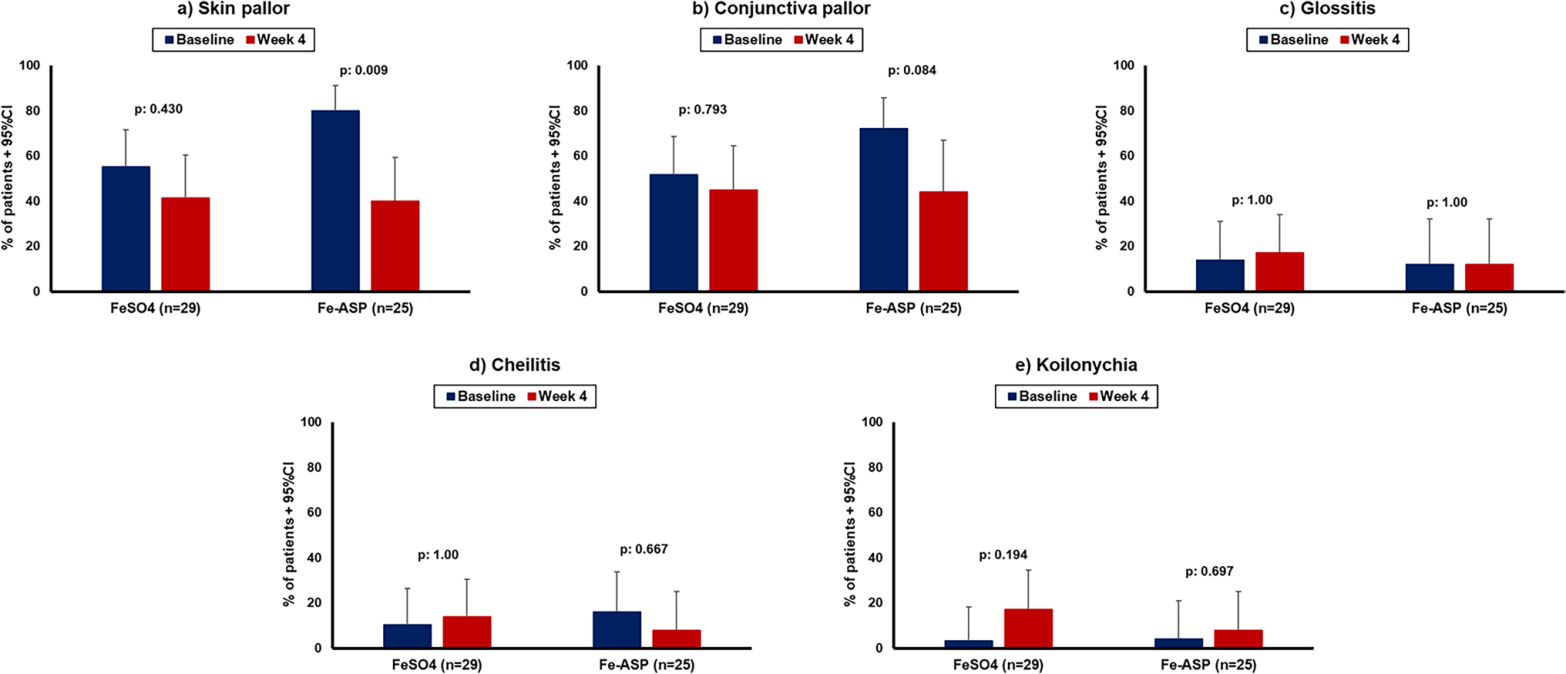
Impact of iron supplementation on iron deficiency anemia (IDA)-related physical signs. The presence of five physical signs of IDA (skin pallor, pallor at the conjunctiva, glossitis, cheilitis, and koilonychia) was evaluated at baseline and by week 4 by the same evaluators. The rates of patients presenting with these signs are provided; the respective *p*-values of comparisons are also shown. Abbreviations: CI, confidence interval; Fe-ASP, iron conjugated to *N*-acetyl-aspartylated derivative of Casein; FeSO_4_, ferrous sulfate

The predefined secondary endpoints by week 4 did not differ between the two groups (Table 2). However, the global improvement score by week 4 was significantly different in the Fe-ASP group than in the FeSO_4_ group (Fig. 3). This score showed that the odds for a worse outcome in the Fe-ASP group was 0.35 compared to the FeSO_4_ group. The rate of skin pallor was significantly decreased at week 4 in the Fe-ASP group but not in the FeSO_4_ group (Fig. 4). Circulating hepcidin was increased from the baseline visit to the next visits in eight of 29 patients (27.6%) of the FeSO_4_ group and in one of 25 patients (4.0%) of the Fe-ASP group (*p*: 0.028) (Supplementary Fig. 1).

**Table 2:** Outcomes of the study by week 4

	Intent-to-treat population				Per protocol population			
Variable*	FeSO_4_	Fe-ASP	Difference	95% CIs	FeSO_4_	Fe-ASP	Difference	95% CIs
Primary endpoint								
Absolute change of Hb, g/dl, mean (SD)	0.77 (1.44)	0.83 (1.59)	−0.06	−0.89; 0.77	0.96 (1.43)	0.89 (1.58)	0.07	−0.79; 0.94
% change of Hb, mean (SD)	10.3 (18.9)	10.9 (20.2)	−0.68	−11.4; 10.1	12.8 (19.1)	11.8 (20.3)	1.08	−10.3; 12.4
Secondary endpoints								
Absolute change of RBC, × 10^6^ cells/mm^3^, mean (SD)	0.24 (0.40)	0.32 (0.65)	−0.09	−0.38; 0.21	0.30 (0.37)	0.33 (0.66)	−0.03	−0.34; 0.27
% change of RBC, mean (SD)	6.6 (11.0)	9.6 (20.2)	−2.99	−11.8; 5.86	8.3 (10.3)	9.9 (20.6)	−1.7	−11.0; 7.6
Absolute change of reticulocytes, × 10^3^ cells/mm^3^, mean (SD)	−42.1 (66.9)	120.7 (416.8)	−168.8	−474.8; 149.1	−47.2 (709.9)	105.9 (419.0)	−153.1	−489.9; 183.7
% change of reticulocytes, mean (SD)	−3.94 (29.60)	12.37 (32.27)	−16.3	−33.4; 0.75	−4.4 (31.4)	11.6 (32.7)	−16.0	−34.4; 2.4
Absolute change of MCV, fl, mean (SD)	2.53 (5.25)	3.39 (3.84)	−0.86	−3.69; 1.97	2.54 (5.37)	3.42 (3.63)	−0.88	−3.85; 2.08
% change of MCV, mean (SD)	3.8 (7.6)	3.5 (5.4)	0.3	−3.3; 3.9	4.1 (7.9)	3.5 (5.2)	0.6	−3.3; 4.5
Absolute change of MCH, g/dl, mean (SD)	−1.67 (12.29)	0.34 (1.28)	−2.01	−7.59; 3.57	−1.75 (12.55)	0.58 (1.09)	−2.33	−8.35; 3.68
% change of MCH, mean (SD)	0.8 (16.7)	1.1 (5.5)	−0.3	−7.2; 6.6	1.5 (17.4)	1.9 (4.9)	−0.3	−7.7; 7.1
Absolute change of fatigue, mm, mean (SD)	−12.7 (22.2)	−16.8 (32.1)	4.14	−11.3; 19.6	−17.71 (15.22)	−17.14 (32.88)	−0.56	−15.65; 14.52
% change of fatigue, mean (SD)	−15.4 (53.8)	−36.4 (34.2)	20.9	−5.6; 47.4	−27.7 (22.9)	−37.4 (34.7)	9.7	−7.7; 27.2
At least one of nausea, epigastric comfort, vomiting, *n* (%)	14 (48.3)	9 (36.0)	12.3	−13.5; 35.7	10 (40.0)	9 (37.5)	2.5	−23.3; 27.8

*Every variable is calculated as a change per 40mg of elementary Fe

Abbreviations: *Fe-ASP*, iron aspartyl-casein; *FeSO*_*4*_, iron sulfate; *Hb*, hemoglobin; *MCH*, mean corpuscular hemoglobin; *MCV*, mean corpuscular volume; *mm*, millimeter; *n*, number of patients; *RBC*, red blood cell count; *SD*, standard deviation

No difference between the two groups of treatment was found for the absolute and relative changes of Hb, RBC count, and reticulocytes at week 12 (Supplementary Fig. 2); for the absolute changes of ferritin of both weeks 4 and 12 (Supplementary Fig. 3); and for the intensity of fatigue at week 12 (Supplementary Fig. 4). Eight of 18 patients (44.4%) treated with FeSO_4_ and 6 of 15 patients (40.0%) treated with Fe-ASP had at least one of symptoms of nausea, vomiting or epigastric comfort at week 12 (*p*: 1.00)

No differences were found regarding the safety profile between the two groups (Table 3).

**Table 3:** Serious and non-serious treatment-emergent adverse events (TEAEs)

	FeSO_4_ (*n*= 29)	Fe-ASP (*n*= 25)	*p*-value
Total serious TEAEs, *n* (%)	5 (17.2%)	6 (24.0)	0.736
Hospital admission due to abdominal pain, *n* (%)	5 (17.2%)	6 (24.0)	0.736
Total non-serious TEAEs, *n* (%)	5 (17.2)	7 (28.0)	0.513
Abdominal pain, *n* (%)	1 (3.4)	1 (4.0)	1.00
Constipation, *n* (%)	0 (0)	1 (4.0)	0.463
Headache, *n* (%)	3 (10.3)	2 (8.0)	1.00
*Helicobacter pylori* gastritis, *n* (%)	1 (3.4)	1 (4.0)	1.00
Sinus tachycardia, *n* (%)	0 (0)	1 (4.0)	0.463
Fungal esophagitis, *n* (%)	0 (0)	1 (4.0)	0.463

## Discussion

The ACCESS trial showed that the new formulation of Fe-ASP was non-inferior to the traditional oral iron supplementation with FeSO_4_ for the achievement of the primary endpoint, i.e., the increase of baseline Hb. The ACCESS study aimed to document the global benefit of iron supplementation on a constellation of the main hematological indexes demonstrating the efficacy of treatment, i.e., change of Hb and of RBC (final result of erythropoiesis) and change of reticulocytes (indication of erythropoiesis reaction). Analysis showed that the odds for these indexes to be improved were greater with Fe-ASP than with FeSO_4_. The study was not powered to document any differences in IDA-related physical findings, GI-related adverse events, and fatigue. As a consequence, any observed statistical differences between groups should be interpreted with caution.

Iron supplementation is the mainstay of the management of IDA. Oral FeSO_4_ is the most commonly studied formulation. In a meta-analysis of 5 trials, more than 78% of participants achieved 1 g/dl of increase of Hb within the first 14 days [17]. This treatment response is within the range of the Hb increase reported in the ACCESS trial. However, it is reported that the extent of the Hb increase relies on the cause of loss of iron, and it is higher among post-partum women. This reported observation is also compatible with the percentage of patients who stopped the study group and who were treated with one intravenous iron formulation.

With the studied formulation of conjugated iron Fe-ASP, it is anticipated that iron is converted to a smaller extent in the stomach into insoluble salts. In this way, more iron reaches the duodenum to become absorbed whereas GI side effects are less often. The current study did not show any major impact of the use of this formulation on the GI adverse effect. There are four clinical studies in a small patient population where Fe-ASP was prospectively administered for 15 to 60 days in patients with IDA. Efficacy was compared to patients treated with ferrous gluconate. In three of these studies, concentrations of hemoglobin (Hb) in blood were greater at the end of treatment with Fe-ASP than with ferrous gluconate. However, oral Fe-ASP supplementation had superior GI tolerability in 96.6% of treated patients compared to 89.2% of patients treated with comparator formulations [13]. Animal studies have shown that casein itself primes the expression of enzymes that facilitate the absorption of iron across the duodenal mucosa [18]. However, in a recent randomized trial in healthy women, the intestinal absorption of iron did not differ between those who were treated with one casein-ion formulation and those treated with FeSO_4_ [19].

The current study has one main strength and three limitations. The main strength is the pragmatic nature of the trial. Study participants were relatively aged (mean age of both groups above 60 years) with substantial comorbidities. Enrolment was done using strict criteria of substantial decrease of Hb, RBC count, and ferritin. It may be conceived that a study investigating the effect of oral iron supplementation should enroll younger people without comorbidities and pregnant women all of which do not present with limitations of iron absorption. However, study participants have the characteristics of patients routinely managed in the clinical setting. The main study limitations are the single-center design and the limited number of participants. Although the study was powered for non-inferiority, the superior efficacy of Fe-ASP over FeSO_4_ in both the global score of improvement and the physical signs of IDA introduces the need for a trial powered for superiority. The score taking into consideration the change of Hb, of RBC count, and of reticulocytes has not been described elsewhere, is novel, and mandates validation. The 12-h oral iron administration may also be considered a study limitation. The study was designed in 2017, and the decision to treat every 12 h was taken based on previous trials suggesting the administration of oral iron supplements two or three times daily [15, 20]. Oral iron supplementation increases the production of hepcidin by the liver which in turn limits the intestinal absorption of iron [10]. As such, it is suggested that iron oral supplements are better administered every 24 or even 48 h^2^. The Fe-ASP formulation is designed to minimize the GI adverse events and have better intestinal absorption. Follow-up measurements in our trial showed that hepcidin was not increased among patients treated with Fe-ASP.

ACCESS is the most recent clinical trial which is evaluating the treatment efficacy of one Fe-ASP formulation in IDA. Fe-ASP was not inferior to FeSO_4_ for the primary endpoint of the Hb change. Some significant findings of the administration of Fe-ASP over FeSO_4_ on the physical signs of IDA and on the global hematology index need confirmation with bigger trials.

## Supplementary information

ESM 1
