# Supplementary material for: An active new formulation of iron carried by aspartyl casein for iron-deficiency anemia: results of the ACCESS trial
Source: Ann Hematol. 2023 Apr 6;102(6):1341–9. doi: 10.1007/s00277-023-05197-3 (PMC10182142; doi:10.1007/s00277-023-05197-3)
Supplement: Supplementary file 1 — ESM 1 [file 277_2023_5197_MOESM1_ESM.docx]

**An active new formulation of iron carried by aspartyl casein for iron-deficiency anemia: results of the ACCESS trial**

**Maria Tsilika^1^, John Mitrou^1^, Nikolaos Antonakos^1^, Ioulia K. Tseti^2^,**

**Georgia Damoraki^1^, Konstantinos Leventogiannis^1^,**

**Evangelos J. Giamarellos-Bourboulis^1^**

**^1^4^th^ Department of Internal Medicine, National and Kapodistrian University of Athens, Medical School, Greece;**

**^2^UNI-PHARMA SA, Kifissia, Greece**


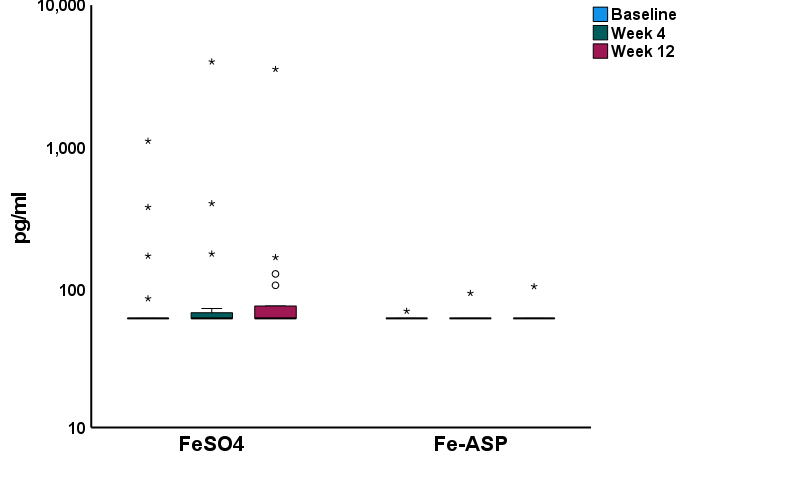


**Supplementary Figure 1 Impact of iron supplementation on hepcidin plasma levels**

Results are presented as boxplots. Circles denote outliers and casterisks denote extremes.

Abbreviations Fe-ASP: iron conjugated to *N*-acetyl-aspartylated derivative of Casein; FeSO_4_: iron sulfate


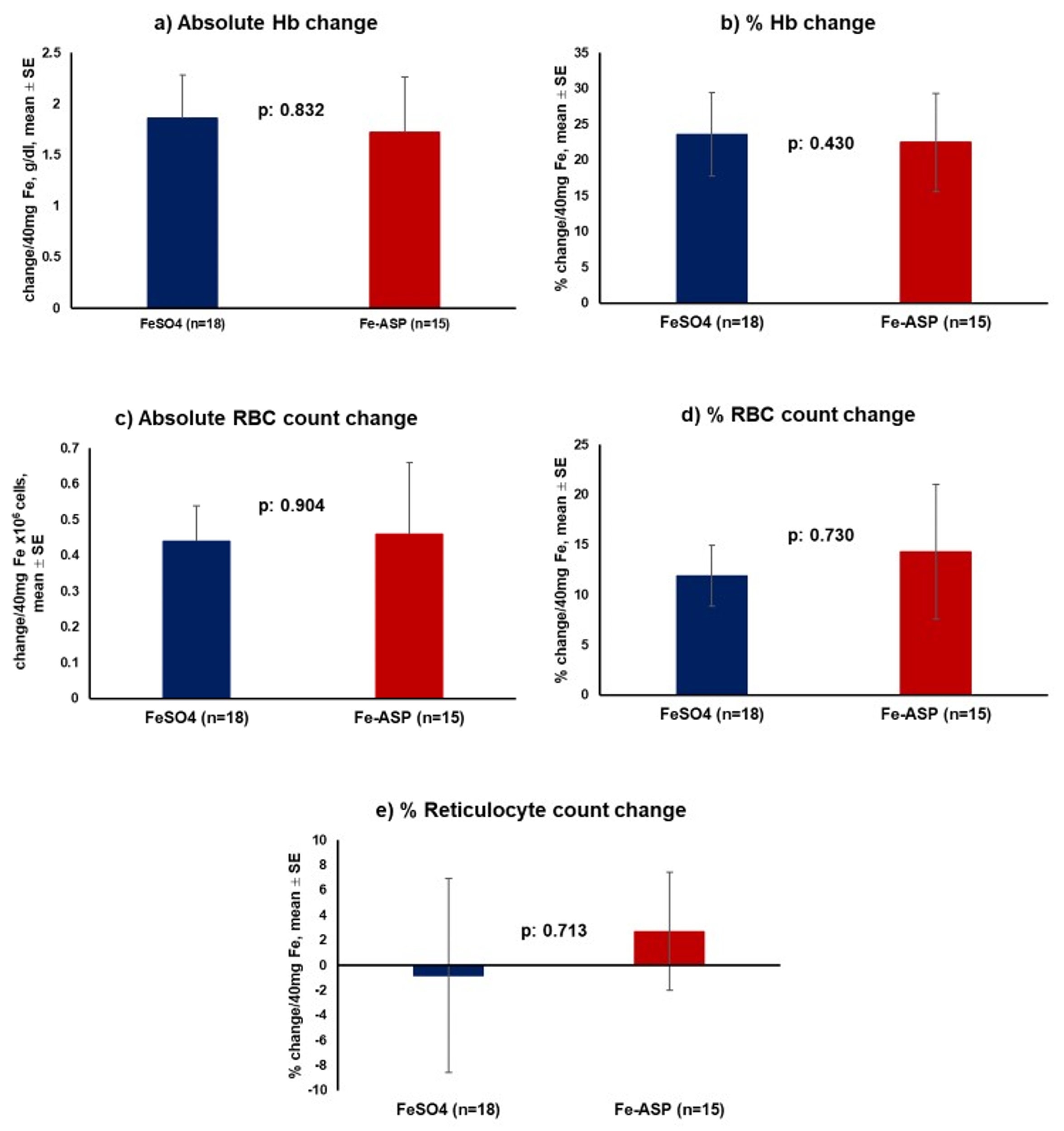


**Supplementary Figure 2 Impact of iron supplementation on hemoglobulin, red blood cell count and reticulocyte by week 12**

Panels show the absolute (a) and relative (b) changes of hemoglobin (Hb) from baseline; the absolute (c) and relative (d) changes of the red blood cell (RBC) count from baseline; and the relative (e) changes of the reticulocyte count from baseline. All values are adjusted per 40mg of Fe supplementation. The p-values of comparisons are also shown.

Abbreviations Fe-ASP: iron conjugated to *N*-acetyl-aspartylated derivative of Casein; FeSO_4_: iron sulfate; OR: odds ratio; SE: standard error


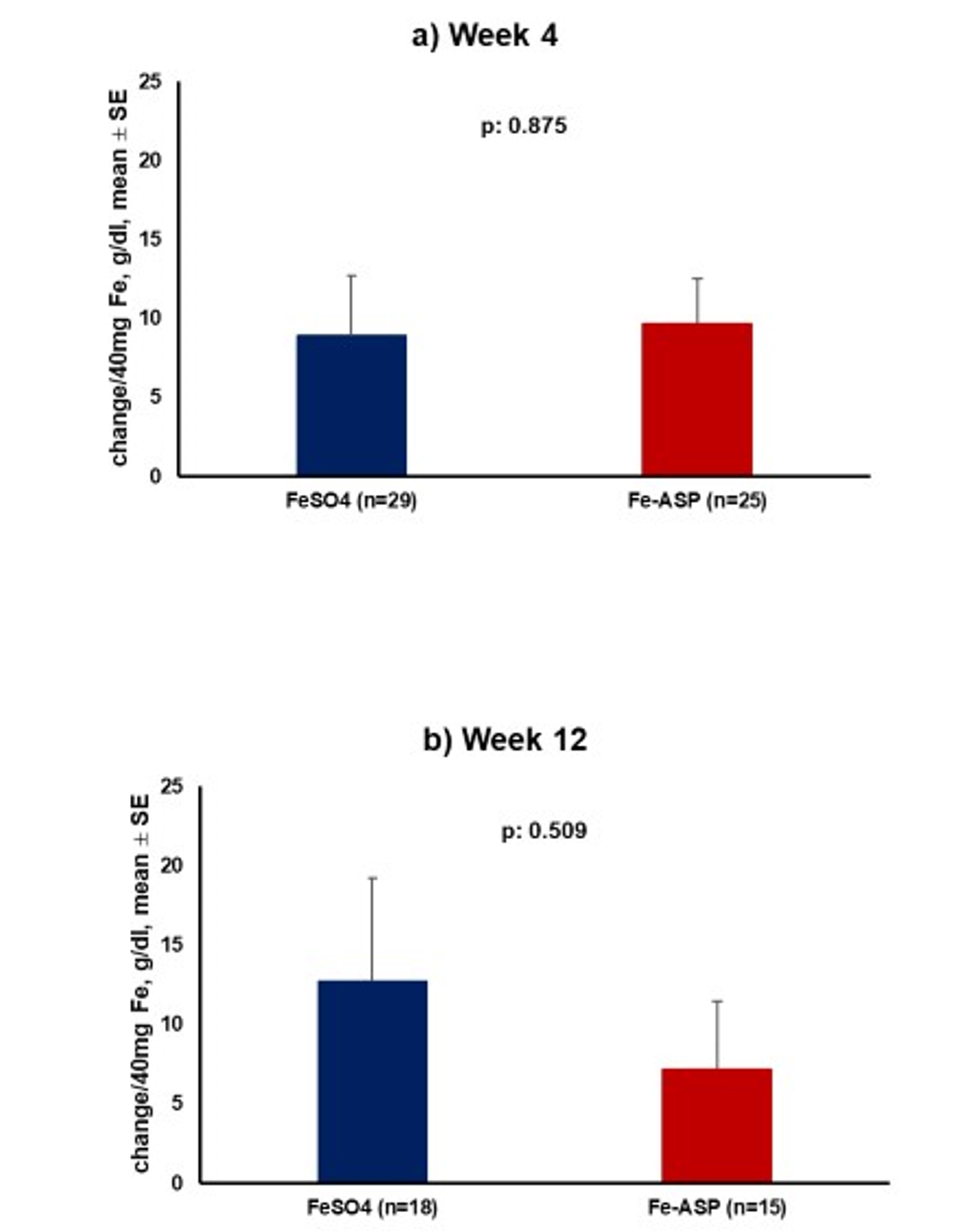


**Supplementary Figure 3 Impact of iron supplementation on ferritin**

Panels show the absolute changes of ferritin ate week 4 (a) and week 12 (b) from the start of the study drug. All values are adjusted per 40mg of Fe supplementation. The p-values of comparisons are also shown.

Abbreviations Fe-ASP: iron conjugated to *N*-acetyl-aspartylated derivative of Casein; FeSO_4_: iron sulfate; OR: odds ratio; SE: standard error


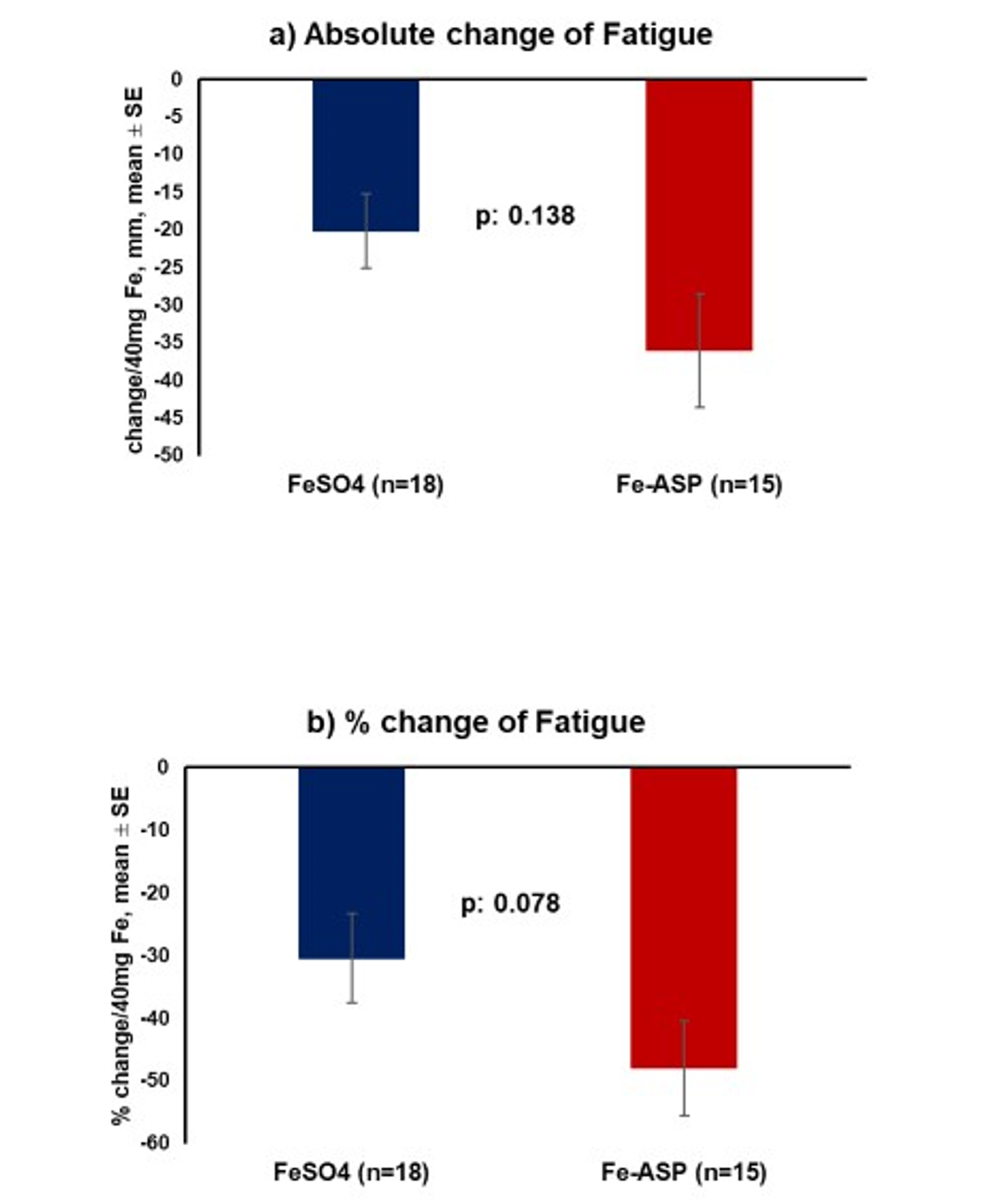


**Supplementary Figure 4 Impact of iron supplementation on fatigue intensity at week 12**

The absolute and relative changes of the visual analogue scale of the intensity of the fatigue syndrome at week 12 after start of the study drug is provided. All values are adjusted per 40mg of Fe supplementation.

The p-values of comparisons are also shown.

Abbreviations Fe-ASP: iron conjugated to *N*-acetyl-aspartylated derivative of

Casein; FeSO_4_: iron sulfate; SE: standard error
